# *Yersinia pseudotuberculosis* bacteraemia with splenic abscesses: a case report

**DOI:** 10.1099/acmi.0.000525.v3

**Published:** 2023-09-25

**Authors:** Rahel Tefera Zewude, Aleksandra Stefanovic, Zersenay Alem

**Affiliations:** ^1^​ Division of Infectious Diseases, Department of Medicine, University of Toronto, Toronto, Canada; ^2^​ Division of Medical Microbiology, Department of Pathology and Laboratory Medicine, University of British Columbia, Vancouver, Canada; ^3^​ Microbiology and Virology Laboratory, St. Paul’s Hospital, Providence Health Care, Vancouver, Canada; ^4^​ Department of Medical Imaging, University of Toronto, Toronto, Canada; ^5^​ Department of Medical Imaging, Sunnybrook Health Sciences Centre, Toronto, Canada

**Keywords:** bacteraemia, pseudotuberculosis, splenic abscesses, *Yersinia*

## Abstract

**Introduction.:**

*
Yersinia pseudotuberculosis* has been known to cause a variety of clinical manifestations ranging from mild enteric illness to bacteraemia with septic shock and extraintestinal abscesses. Patients with liver disease and iron overload are at risk of more severe disease manifestations.

**Case Report.:**

A middle-aged male with chronic alcohol use disorder presented with confusion and jaundice, with ascites and asterixis noted on examination. His blood work was remarkable for neutrophilic leukocytosis, elevated liver enzymes and lactate. An abdominal computed tomography scan revealed splenic microabscesses and a cirrhotic liver. *Yersinia pseudotuberculosis* was recovered from his blood cultures and he was treated with ceftriaxone following susceptibility results.

**Conclusion.:**

*
Y. pseudotuberculosis* should be considered in the differential diagnosis of splenic or other extraintestinal microabscesses particularly in patients with chronic liver disease.

## Data Summary

Accession number for blood culture: T2200616. This was a clinical sample and therefore it was not uploaded to any database.

## Background

*Yersinia pseudotuberculosis* is a Gram-negative bacillus from the family *Yersiniacae*. Similar to another member of the family, *Yersinia enterocolitica, Y. pseudotuberculosis* most commonly presents with enteric illness [1].

The sources of infection with *Y. pseudotuberculosis* include contaminated food such as dairy products and vegetables or contaminated water [2]. Several animals including birds, dogs, rodents, rabbits, deer and farm animals can serve as reservoirs [1, 2]. Direct contact with these animals has also been described as a mode of acquisition [1, 2]. Community outbreaks secondary to consumption of contaminated lettuce, carrots and water have been reported in Finland, Japan, Russia and Canada [3, 4].

Although it often presents as mild gastroenteritis, several other clinical manifestations have been reported with *Y. pseudotuberculosis* [1]. Pseudoappendicitis with mesenteric lymphadenitis is a well-described phenomenon and many patients in the 20th century underwent appendectomies due to erroneous diagnosis after presenting with right lower quadrant abdominal pain [5]. Bacteraemia is rare and when it occurs it has been associated with abscesses in the spleen, liver, kidneys and lung with a granulomatous appearance that mimics tuberculosis [5, 6]. Although it is commonly a self-limiting illness in immunocompetent patients, septicaemia with *Y. pseudotuberculosis* infection is thought to carry a fatality rate that exceeds 75 % without use of antibiotics [2].

Here we present a case of *Y. pseudotuberculosis* bacteraemia with splenic abscesses in a patient with previously undiagnosed liver cirrhosis.

## Case report

### Clinical presentation

A middle-aged male patient with chronic alcohol use disorder and a recent trip to Mexico presented with a 2 week history of progressive confusion and jaundice.

He complained of abdominal pain in the previous week and increasing abdominal girth for the prior 2 weeks. He did not have any diarrhoea or frank abdominal pain, fevers or chills. He denied any respiratory, cardiac or urinary symptoms.

The patient did not have any immune compromising conditions and he was not taking any home medications. However, he had chronic alcohol use disorder with 25–50 oz (740–1479ml) of vodka per day for over 10 years. He also had a unilateral eye prosthesis following globe rupture from glass trauma 20 years previously.

He recently returned from a 2 week trip to Mexico where he denied consuming any unpasteurized milk, raw meat or raw vegetables. The patient recalled having barbecued pork meat that he thought was well cooked.

### Physical examination

The patient appeared confused but not in any distress in the initial assessment. His blood pressure was 104/72 mmHg, pulse 124 beats per minute, temperature 36.5 °C and respiration rate of 18 breaths per minute with oxygen saturation of 93 % on room air. His examination was notable for mild asterixis, jaundice as well as soft, non-tender abdomen with mild ascites.

### Investigations

#### Initial laboratory investigations

His blood count showed a white blood cell count of 17.7×10^9^ l^–1^, haemoglobin of 129 g l^–1^, platelets of 63×10^9^ l^–1^, International normalized ratio of 1.5 and C-reactive protein of 67.5 mg l^−1^ (Table 1). His total bilirubin was 126 µmol l^–1^ with direct bilirubin of 79 µmol l^–1^. The remaining liver function tests showed alanine transaminase of 37 U l^–1^, alkaline phosphatase of 187 U l^–1^, gamma glutamyl transferase of 170 U l^–1^, albumin of 23 g l^–1^ and lactate of 3 mmol l^–1^ (Table 1).

**Table 1.** Laboratory reference values

Laboratory parameter	Normal reference range
Albumin	38–50 g l^–1^
Alkaline phosphatase (ALP)	40–150 U l^–1^
Alanine transaminase (ALT)	7–40 U l^–1^
C-reactive protein (CRP)	≤11.0 mg l^−1^
Direct bilirubin	2–9 µmol l^–1^
Gamma glutamyl transferase (GGT)	15.0–85.0 IU l^–1^ (male) 5.0–55.0 IU l^–1^ (female)
Haemoglobin	120–160 g l^–1^
International normalized ratio (INR)	0.9–1.2
Lactate	0.5–2.2 mmol l^–1^
Platelets	150–400×10^9^ l^–1^
Total bilirubin	3–17 µmol l^–1^
Transferrin saturation	0.20–0.50
White blood cell count	4–11.0×10^9^ l^–1^

#### Radiographic investigations

**Fig. 1.**
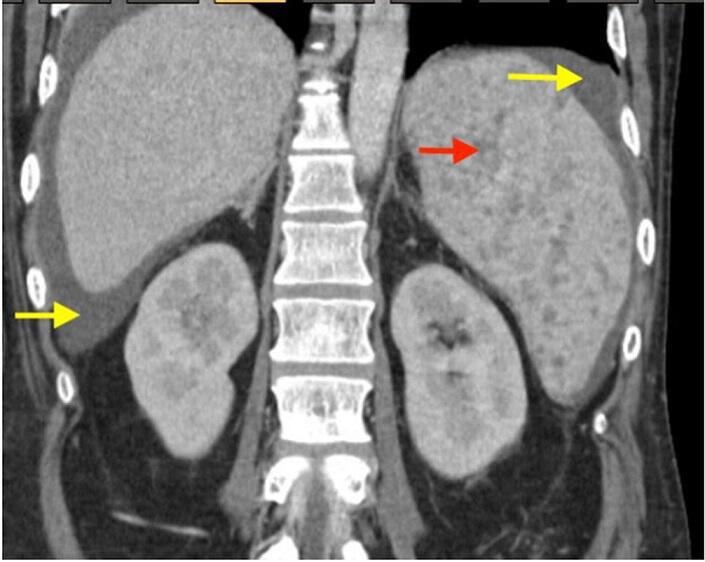
CT scan of the abdomen with contrast, coronal view. Numerous hypoattenuating small nodules are seen throughout the spleen (red arrow). These imaging features are non-specific, but are compatible with microabscesses. The liver is nodular, and the spleen is moderately enlarged. Perihepatic and perisplenic ascites (yellow arrows) are present, in keeping with underlying cirrhosis. There are no obvious hepatic masses or abscesses.

**Fig. 2.**
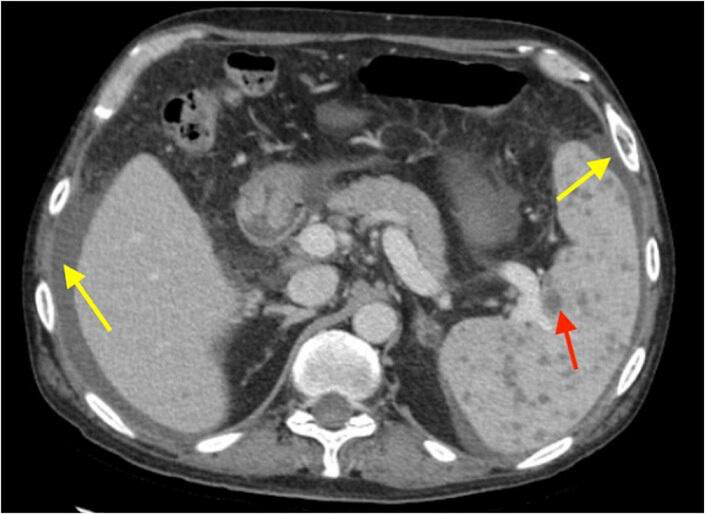
CT scan of the abdomen with contrast, axial view. Numerous hypoattenuating small nodules are seen throughout the spleen (red arrow). These imaging features are non-specific, but the radiographic appearance is compatible with microabscesses. The liver is nodular, and the spleen is moderately enlarged. Perihepatic and perisplenic ascites (yellow arrows) are present, in keeping with underlying cirrhosis. There are no obvious hepatic masses or abscesses.

A computed tomography (CT) scan of the abdomen revealed splenomegaly with innumerable hypodense lesions in the spleen consistent with microabscesses, nodular liver and moderate ascites (Figs 1 and 2).

#### Microbiology investigations

**Fig. 3.**
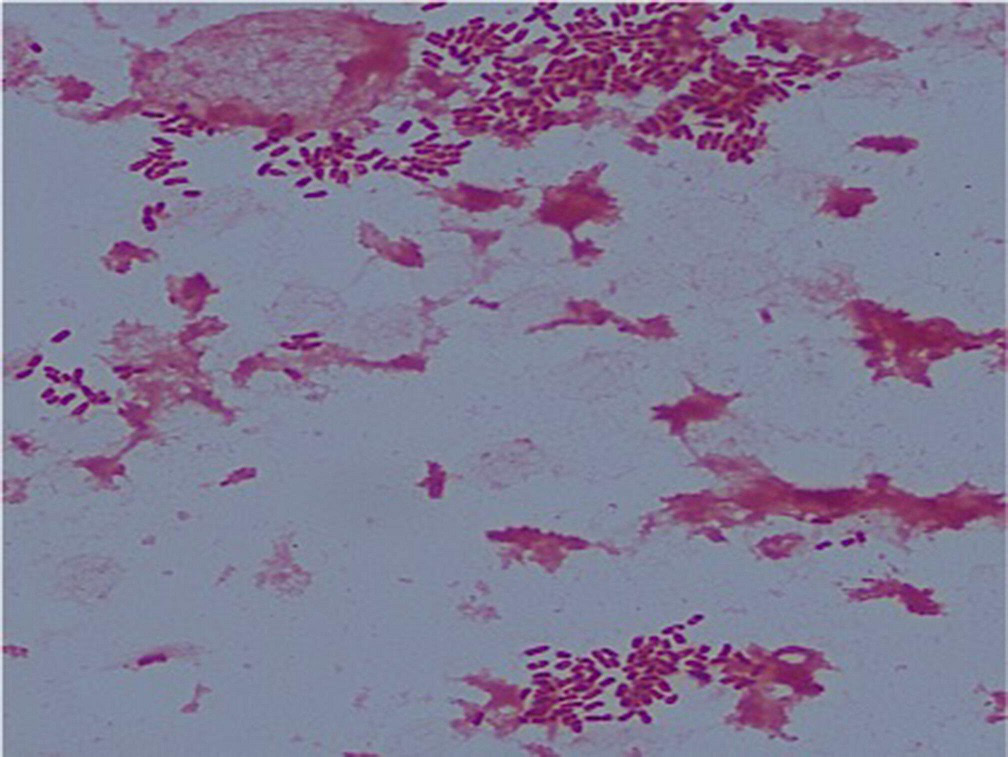
Gram stain of blood culture sample. Short Gram-negative bacilli are seen under light microscopy; 1000× magnification.

Blood cultures grew Gram-negative coccobacilli in all four bottles. Gram staining showed short Gram-negative bacilli (Fig. 3). The bacterium was identified as *Y. pseudotuberculosis* by Vitek MS (bioMérieux) matrix-assisted laser-desorption/ionization time-of-flight (MALDI-TOF) MS, a fully validated method for routine bacterial identification in our clinical laboratory [7]. Mass spectra profiles were analysed using the commercial Vitek MS database (MS-ID version v.3.2) and a high confidence score of 100 % was obtained.

Antibiotic susceptibility testing (AST) was performed using the Kirby–Bauer disc diffusion method. *Y. pseudotuberculosis* was susceptible to ampicillin, cefazolin, ceftriaxone, piperacillin-tazobactam, ciprofloxacin, trimethoprim-sulfamethoxazole, gentamicin and tobramycin. Ciprofloxacin susceptibility was confirmed by the gradient diffusion method (i.e. Etest; bioMérieux) with MIC of 0.032 µg ml^−1^. AST results were interpreted as per the Clinical and Laboratory Standards Institute (CLSI) guidelines for *Enterobacterales* [8].

#### Treatment and follow-up

On admission, the patient was empirically started on intravenous piperacillin-tazobactam which cleared his bacteraemia within 48 h. Six days after admission, he underwent a diagnostic and therapeutic paracentesis. His peritoneal fluid analysis was consistent with bacterial peritonitis with white blood cell count of 1176×10^6^ l^–1^ and 50 % neutrophils. However, as this was collected after 6 days of antibiotics, peritoneal fluid Gram stain showed 2+ mononuclear cells with no organisms seen and there was no growth on culture.

During his hospital stay, the patient was also found to have bilateral redness of his eyes with associated occasional discharge. He was evaluated by ophthalmology on day 9 of admission. At the time of his ophthalmology examination, however, his symptoms had resolved with no further eye redness or discharge. Both his native eye and contralateral eye socket with an eviscerated globe appeared healthy on anterior and posterior segment examination by ophthalmology with no findings of conjunctivitis or endophthalmitis.

The patient’s confusion, thought to be due to hepatic encephalopathy, resolved with lactulose treatment. Once susceptibilities became available, he was switched to intravenous ceftriaxone to complete a total of 14 days of parenteral antibiotic treatment. His liver enzymes also improved markedly a week after his admission and the patient was subsequently discharged home.

## Discussion

*Y. pseudotuberculosis* is a non-lactose-fermenting Gram-negative bacillus with characteristic bipolar staining. It is a urease-positive but indole- and oxidase-negative organism that is motile at room temperature [9]. In addition to MALDI-TOF MS, it can be identified by automated biochemical methods such as Vitek2 ID (bioMérieux).

Since its first isolation in 1883, *Y. pseudotuberculosis* has been reported across the globe with a range of clinical manifestations [10]. A *Y. pseudotuberculosis* outbreak associated with homogenized milk from a milk plant occurred in Canada in 1998 [11]. This outbreak reported 74 laboratory-confirmed cases in the province of British Columbia, with isolation of *Y. pseudotuberculosis* from stool, blood or mesenteric nodes [11]. Currently available reports of *Y. pseudotuberculosis* infections describe great variability in the extent of disease severity. Severe manifestations include bacteraemia with or without septic shock, hepatic or splenic abscesses, and rarely facet joint infections and septic arthritis [2, 10].

Beyond acute infectious presentations of bacteraemia and extraintestinal abscesses such as splenic abscesses, *Y. pseudotuberculosis* is also known for its immunological complications. Immunological complications of *Y. pseudotuberculosis* infection include erythema nodosum, glomerulonephritis, reactive arthritis, isolated conjunctivitis, iritis and onset of Kawasaki disease [1]. In our patient, his new-onset bilateral redness of his eyes raised a concern for an immunological complication such as conjunctivitis. However, these remain unproven given the complete resolution of these symptoms at the time of his ophthalmological assessment. Another complication, Far East scarlet like fever (FESLF), is a severe systemic inflammatory manifestation secondary to hypervirulent strains of *Y. pseudotuberculosis* first described in Russia in 1959 [5, 12]. FESLF presents with fever, desquamating rash, variable neurological and gastroenterological symptoms, and rarely cardiovascular symptoms such as arrhythmias [5].

Several patient risk factors associated with severe and systemic *Y. pseudotuberculosis* infections have been described. These include iron overload disorders such as haemochromatosis, cirrhosis, thalassaemia, and haemolytic anemias in addition to immunosuppression [2, 13]. Iron overload disorders create a favourable environment for *Y. pseudotuberculosis* because of its ability to bind iron through secondary metabolites called siderophores [13]. Iron is essential for bacterial growth and is often sequestered in live mammalian hosts becoming inaccessible to many bacteria [14, 15]. Similar to other members of the family *Yersiniace*, *Y. pseudotuberculosis* scavenges iron by using siderophores such as yersiniabctin (Ybt) with high affinity for ferric (Fe^3+^) ions present in mammalian hosts [13]. As a result of its efficient iron scavenging systems, *Y. pseudotuberculosis* can effectively grow and cause severe illnesses such as bacteraemia and extraintestinal abscesses, with greater mortality in patients with iron overload disorders such as liver disease and haemochromatosis [16]. The ability of *Y. pseudotuberculosis* to sense iron availability and oxidative stress has been proposed as the rationale for more extraintestinal dissemination in hosts with iron overload as *Y. pseudotuberculosis* would be more likely to cross the intestinal barrier into deeper organs such as the liver and spleen [17].

In our patient, his presenting symptoms of jaundice, confusion and abdominal girth were secondary to decompensated liver cirrhosis rather than *Y. pseudotuberculosis* bacteraemia. However, his liver cirrhosis and severe alcohol use disorder, which resulted in a state of iron overload, probably led to a more severe and invasive presentation of *Y. pseudotuberculosis* infection with bacteraemia and splenic abscesses. Both severe alcohol use and liver cirrhosis have been shown to affect iron metabolism by increasing iron stores at the liver parenchyma and reticuloendothelial system [18, 19]. Notably, our patient had an elevated transferrin saturation of 0.64 suggestive of increased iron stores although his ferritin was not assessed (Table 1).

Interestingly, our patient presented with bacteraemia associated with splenic microabscess without any hepatic abscesses. Prior to identification of *Y. pseudotuberculosis* in his blood cultures, the radiographic differential diagnosis for his splenic microabscesses included fungal infection, bacterial infection with atypical bacteria such as *Salmonella* spp*.* and *Mycobacterium* spp*.,* granulomatous disease with sarcoidosis or lymphoma.

Hepatosplenic dissemination of *Y. pseudotuberculosis* was previously thought to result from the ability of this species to be translocated by specialized intestinal epithelial cells into lymphoid Peyer’s patches of the ileum [20]. *Y. pseudotuberculosis* possesses a protein on its outer membrane, invasin, that can bind to intestinal epithelial cells, and this was thought to facilitate the uptake and dissemination of *Y. pseudotuberculosis* into Peyer’s patches and subsequently into mesenteric lymph nodes that drain into the bloodstream [21, 22]. Although this hypothesis had been widely accepted, recent studies with mice have demonstrated that extraintestinal spread of *Y. pseudotuberculosis* is complex and involves alternative routes such as movement into the portal vein system from the intestinal epithelium and reaching the liver and spleen through the pathway of the portal vein rather than by lymphatic drainage [20]. Furthermore, in Peyer’s patch-deficient mice, it was shown that dissemination of *Y. pseudotuberculosis* into the spleen, liver and mesenteric lymph nodes was not reduced, calling into question the role of Peyer’s patches in hepatosplenic dissemination [22].

Since the first reported human case of *Y. pseudotuberculosis* bacteraemia in 1911, there have been seven reports of splenic abscesses [10]. Four of these were isolated splenic abscesses while the others occurred in conjunction with hepatic abscesses and in one case along with renal abscesses [7, 23–26]. It is worth noting that four out of the seven cases were fatal [10]. All of the four fatal cases were described before 1943, prior to mass production of penicillin for clinical application, and the patients in these cases did not receive any antimicrobial therapy [10, 27].

In previously reported cases of bacteraemia due to *Y. pseudotuberculosis *, treatments with penicillin, first- and third-generation cephalosporins, fluoroquinolones and carbapenems have been used with successful clinical outcomes [2]. The optimal antimicrobial choice, however, has not been formally established yet.

## Conclusion

*Y. pseudotuberculosis* infection represents an important differential diagnosis in patients with splenic abscesses. Exposure to contaminated water, vegetables and dairy products should be assessed in such cases to determine modes of acquisition of *Y. pseudotuberculosis* infection. Immunosuppression and iron overload disorders such as haemochromatosis and chronic liver disease are important risk factors for severe and disseminated *Y. pseudotuberculosis* infections where it carries a high fatality rate in these patient populations.

Manifestations of *Y. pseudotuberculosis* can range from mild gastroenteritis to sepsis and abscesses in the spleen, liver and lung. In addition to treating yersiniosis with antimicrobials, it is also important to monitor patients for immunological complications such as erythema nodosum, glomerulonephritis, conjunctivitis and Kawasaki disease.
